# The Use of Smart Devices by Care Providers in Emergency Departments: Cross-Sectional Survey Design

**DOI:** 10.2196/13614

**Published:** 2019-05-05

**Authors:** Mohamad Alameddine, Hussein Soueidan, Maha Makki, Hani Tamim, Eveline Hitti

**Affiliations:** 1 American University of Beirut Faculty of Health Sciences Department of Health Management and Policy Beirut Lebanon; 2 American University of Beirut Evidence Based Health Management Unit Beirut Lebanon; 3 American University of Beirut Faculty of Medicine Department of Emergency Medicine Beirut Lebanon; 4 American University of Beirut Faculty of Medicine Department of Internal Medicine Beirut Lebanon

**Keywords:** health personnel, smart phones, emergency departments, healthcare quality, policy

## Abstract

**Background:**

The use of smart devices (SDs) by health care providers in care settings is a common practice nowadays. Such use includes apps related to patient care and often extends to personal calls and applications with frequent prompts and interruptions. These prompts and interruptions enhance the risk of distractions caused by SDs and raise concerns about service quality and patient safety. Such concerns are exacerbated in complex care settings such as the emergency department (ED).

**Objective:**

The objective of this study was to measure the frequency and patterns of SD use among health care providers in the ED of a large academic health center in Lebanon. The perceived consequences of care providers using SDs on provider-to-provider communication and the care quality of patients in the ED were assessed. Additionally, factors associated with the use of SDs and the approval for regulating such use were also investigated.

**Methods:**

The study was carried out at the ED of an academic health center with the highest volume of patient visits in Lebanon. The data were collected using a cross-sectional electronic survey sent to all ED health care providers (N=236). The target population included core ED faculty members, attending physicians, residents, medical students, and the nursing care providers. The regression model developed in this study was used to find predictors of medical errors in the ED because of the use of SDs.

**Results:**

Half of the target population responded to the questionnaire. A total of 83 of 97 respondents (86%) used one or more medical applications on their SDs. 71 out of 87 respondents (82%) believed that using SDs in the ED improved the coordination among the care team, and 71 out of 90 (79%) respondents believed that it was beneficial to patient care. In addition, 37 out of 90 respondents (41%) acknowledged that they were distracted when using their SDs for nonwork purposes. 51 out of 93 respondents (55%) witnessed a colleague committing a near miss or an error owing to the SD-caused distractions. Regression analysis revealed that age (*P*=.04) and missing information owing to the use of SDs (*P*=.02) were major predictors of committing an error in the ED. Interestingly, more than 40% of the respondents were significantly addicted to using SDs and more than one-third felt the need to cut down their use.

**Conclusions:**

The findings of this study make it imperative to ensure the safety and wellbeing of patients, especially in high intensity, high volume departments like the ED. Irrespective of the positive role SDs play in the health care process, the negative effects of their use mandate proper regulation, in particular, an ethical mandate that takes into consideration the significant consequences that the use of SDs may have on care processes and outcomes.

## Introduction

### Background

A smart device (SD) is an electronic tool characterized by its mobility, connectivity, and potential to provide access to internet, geographical location, and relatively independent operation. SDs have the capability to gather information about their users and the environment to optimize users’ experience [1,2]. Most commonly used SDs include smartphones, tablets, smart watches, and wearable devices, among others.

The use of SD in the health care industry is rising and gaining attention [3]. With over 70,000 health and medical SD apps available, their use for clinical practice has become common [4]. A study by Koehler shows that clinical applications include mobile result checking, drug references, clinical decision tools, case management tools, as well as staff scheduling modules, which have made SD usage integral in day to day clinical practice [5].

The use of SDs in the clinical setting, however, is associated with risks and concerns. Risks include potential breaches to patient privacy, as well as cross-contamination concerns related to cleanliness of the devices [5]. The negative impact on physician-patient relationship, as well as interprofessional relationship is another feared consequence [6,7]. Finally, there is growing evidence related to the distracting power of SD, which raises serious questions about the safety implications of use of SD in the health care settings where interruptions are common and the need to reduce error and improve safety is paramount [8].

Decision making in medicine requires significant cognitive focus integrating increasingly complex information from different sources, often under significant time constraints, in settings where interruptions are common. Previous research shows that interruptions are disruptive to care processes and may cause increased risks of error related to patient care, medication management, and communication among health care providers [9]. Emulating the aviation industry that has moved to *sterile cockpits* to reduce distractions, hospitals have started introducing separate medication rooms to reduce errors in mixing and preparing medications [10,11]. There is also increasing interest in creating similar distraction-free spaces for other high-risk activities in health care [12].

Emergency departments (EDs) are settings that are particularly prone to error, and the cases in the ED are often complex and the workflow dynamics and decisions are made under significant time pressures [13]. Furthermore, when compared with the interruptions in the primary care clinics, ED physicians are 3 times more likely to be interrupted [14]. Understanding the patterns of provider utilization of SDs in this high-risk setting and the perceptions of their impact on distraction and safety is an important first step in formulating policy or practicing recommendations for their use in health care settings.

### Objectives

This study investigated self-reported health care providers’ utilization of SDs in the ED and their perception regarding such usage on the patient-provider communication and on the safety of care at the ED of a large academic medical center in Beirut, Lebanon. The outcomes of the study will help in developing effective evidence-based policies to guide the use of SDs in clinical settings. The specific objectives of this study are as follows:

- Investigate the frequency and patterns of SD use among health care providers in the ED of a large academic health center in Lebanon.
- Understand the perceived consequences by care providers of SD use on patients and health care providers.
- Examine the factors associated with the use of SDs.
- Assess the perceptions toward potential SD regulatory policies in the ED of the target facility.

## Methods

### Ethical Approval

The study was approved by the Institutional Review Board (IRB) at the target institution under the protocol number ED.EH.06. The research team carried out the research work associated with this study after obtaining the IRB approval. ED providers were clearly instructed that this was an independent research study and that they had the full autonomy to participate without any rewards or consequences on their employment with the institution.

All participants had to read and sign an informed consent emphasizing that participating in this study was voluntary and that the participants had the full freedom to choose whether to participate or not. The participants were assured that their responses would be kept confidential and anonymous and that no one would have access to the data other than the researchers themselves. The participants were informed that the outcomes of the project would be used to guide the evidence-based use of SDs by health care providers in EDs.

### Study Setting

The study was conducted in the ED of a large academic tertiary care hospital in Lebanon. The ED receives more than 57,000 visits per year and is the busiest ED in Lebanon. The ED includes 2 adult sections that separate patients according to their age and case acuity, and 1 pediatric section that receives all the pediatric cases. The ED is also considered an integral teaching setting in the education of medical students as more than 180 medical students and residents rotate through it every year.

### The Survey Tool

The survey tool included, in addition to other items, questions on demographics, job position, years of experience, frequency of SD use, the types of used SDs, and applications. Using Likert scales, the health care professionals were asked to rate their perceptions toward using SDs, their perceived effect on patient care and team performance, addiction to using the SDs, and their opinion with regard to instituting a code of conduct to regulate the use of SD at the ED.

Adapted from the *Cut Down, Annoyed, Guilty and Eye Opener alcohol use disorders screening test,* the CAGE tool, the research team used a set of questions to measure the addiction score pertinent to using SDs. Similar uses of the CAGE tool have been documented in previous research [15-18]. A respondent with a score higher than 2 positive answers out of the 5 questions was considered to be addicted to using SDs [17].

To enhance content validity, the final survey tool was reviewed by a group of experts including a health human resources management expert, a statistician, and a social scientist. The tool was also shared with international experts for opinion. All feedback was consolidated in the final version of the questionnaire, which was pilot tested on a number of care providers.

### Data Collection

The email addresses of all ED providers were obtained from the ED management (as per the approved IRB protocol). ED providers were initially invited to participate in this study through an IRB-approved invitation email script. The email script included information about the study, the research team, what does it entail to participate, and the full respect for the freedom to participate, as well as the privacy and confidentiality of all answers. Should an invitee agree to participate, they were asked to click the Web-based survey link which started with an electronic consent form that needed to be signed before completing the questionnaire.

During the period January 2017 and September 2017, data were collected from ED health care providers using a 15-min electronic questionnaire on LimeSurvey (GmbH, Survey Services and Consulting, Hamburg, Germany), an electronic surveying platform.

The survey tool was made available only in English as all the target health care professionals were proficient in verbal and written English. A contact email address was provided to the participants in case they had any questions or inquires relevant to the study.

### Study Population

A total of 236 health care providers working at the ED during the period of data collection were approached to participate in the study. Health care providers at the time of the study included 7 core ED faculty, 25 clinical associate faculty members, 53 ED residents, 62 nurses, 37 nursing students, and 52 rotating medical students. The invitation to participate in this study was extended to all health care providers working in the ED.

### Data Analysis

The data retrieved from the LimeSurvey GmbH included 118 responses. The research team discarded 18 records as the respondents had missed more than 25% of the questions that belonged to main scales. After further examination of the dataset, the data collected from nursing students (n=3) had to be removed as such a small sample size rendered representativeness and comparability difficult. The final sample size included 97 records. IBM SPSS version 22 was used for analysis.

The continuous variables were grouped and summarized by frequency number and the same was done in the case of the categorical variables. Descriptive univariate and bivariate analyses were carried out to get insights into the different variables and the association between the dependent and the independent variables was obtained using the Pearson chi-square test. A *P* value less than .05 was considered statistically significant.

To define the predictors of making an error during an ED shift, the research team constructed a stepwise logistic regression model that included the effect of age and missing information as a result of using the SD as the predictors of making an error, while controlling for other variables (age, gender, job position, experience, whether the respondents had missed information because of using their SDs, using the SDs for personal reasons during the ED shift, and the addiction score). A *P* value less than .05 was considered to be statistically significant.

## Results

### Sample Demographics

The response rate in this study was 50%, with a total of 118 responses (out of 236 potential) received. However, after cleanup, 97 responses were eligible for inclusion in the final analysis.

The study sample was relatively young and equally distributed among males and females. The sample was distributed among the professions, including 32 nurses (32/97, 33%), 23 medical students (23/97, 24%), 22 residents (22/97, 23%), and 20 attending physicians (20/97, 20%). The majority of the respondents (82/97, 85%) had completed their training or residency in Lebanon. Furthermore, 19 out of 33 (58%) responded that they had been practicing professionally for more than 5 years, including almost one-third of them (10 out of 33, 30%) with experience over 10 years. Table 1 exhibits the detailed percentage distribution of the demographic and professional background of the survey respondents.

**Table 1:** Distribution of the demographic and professional background.

Demographic and professional background		n (%)
**Age in years (N=97)**		
	<30	61 (63)
	30-39	23 (24)
	40-49	7 (7)
	50-65	6 (6)
**Gender (N=93)**		
	Male	47 (50)
	Female	46 (50)
**Discipline (N=97)**		
	Nurse	32 (33)
	Medical student	23 (23)
	Resident	22 (23)
	Attending physician	20 (21)

### Prevalence of Smart Device Use and Applications

All of the respondents reported owning a SD (Table 2). A total of 86% of the respondents (83 out of 97) used one or more medical applications on their SDs. Among those, 33 out of the 45 respondents (73%) used medical reference applications, such as Medscape, UpToDate, and others. The remaining used other applications including clinical decision applications (18%), pharmacology applications (11%), the ministry of public health application (9%), clinical practice applications (7%), and others (Table 3). The use of nonmedical applications was also very common. Among other applications, 86 out of 94 respondents (92%) used the phone for email access, 81 out of 96 respondents (84%) used their phones for Web access, 84 out of 96 used (88%) the messaging applications (including WhatsApp), 73 out of 96 (76%) used them for phone calls, 72 out of 95 respondents (76%) used the calendar, and 63 out of 95 (66%) used the social media applications (Table 4).

**Table 2:** Use of smart devices and medical applications.

Owning a smart device (N=97)	n (%)
Yes	97 (100)
No	0 (0)

**Table 3:** The most used medical applications.

Most used medical applications (N=45)	n (%)^a^
Medical reference apps	33 (73)
Clinical decision apps	8 (18)
Pharmacology apps	5 (11)
Ministry of public health app	4 (9)
Clinical practice apps	3 (7)
Other applications	4 (8)

^a^The values are not mutually exclusive.

**Table 4:** The most used nonmedical applications.

Most used nonmedical applications	n (%)
Email access (N=94)	86 (92)
Messaging applications (including WhatsApp; N=96)	84 (88)
Web access (N=96)	81 (84)
Phone calls (N=96)	73 (76)
Calendar (N=95)	72 (76)
Social media applications (N=95)	63 (66)

### Reporting of Coworker Distraction and Error When Using the Smart Device in the Emergency Department

A total of 64% of respondents (59 out of 92) had witnessed a sizable proportion of trainees getting distracted when using their SDs for nonwork–related activities at the ED. Furthermore, 50 out 91 respondents (55%) witnessed the same for nurses and 44 out of 90 respondents (49%) witnessed attending physicians getting distracted when using their SDs for nonwork–related activities at the ED (Table 5). Consistently, 37 out of 90 respondents (41%) had acknowledged that using their SDs for nonwork–related purposes distracted them (Table 6).

In addition, 51 out of 93 respondents (55%) reported observing their colleagues having made an error or a near miss as a result of being distracted by their SDs (Table 5). Interestingly, only 15 out of 93 respondents (16%) acknowledged that they did make an error or a near miss as a result of being distracted by their SDs (Table 6).

**Table 5:** Performance in the emergency department when using the smart devices.

Reporting of coworker distraction and error when using the smart device in the emergency department		n (%)
**I have witnessed a trainee (medical student, resident) become distracted when using his or her smart device for nonwork-related activities (N=92)**	
	Yes	59 (64)
	No	33 (36)
**I have witnessed a nurse become distracted when using his or her smart device for nonwork–related activities (N=91)**	
	Yes	50 (55)
	No	41 (45)
**Emergency department** **coworkers made an error or were about to make an error (near miss) as a result of being distracted by the use of the smart device during an** **emergency department** **shift? (N=93)**	
	Yes	51 (55)
	No	42 (45)
**I have witnessed an attending physician become distracted when using his or her smart device for nonwork–related activities (N=90)**	
	Yes	44 (49)
	No	46 (51)

**Table 6:** Reporting of self-distraction and error when using the smart device in the emergency department.

Reporting of self-distraction and error when using the smart device (SD) in the emergency department (ED)		n (%)
**Using my SD for non-work-related purposes distracts me (N=90)**	
	Yes	37 (41)
	No	53 (59)
**Have you ever made an error or were about to make an error (near miss) as a result of being distracted by the use of the smart device during an ED shift? (N=93)**	
	Yes	15 (16)
	No	78 (84)

### Impact of Using Smart Devices in the Emergency Department on the Quality of Care

A total of 71 out of 87 respondents (82%) confirmed that using SDs in the ED had improved care coordination among the health care providers and 71 out of 90 (79%) respondents believed that it was beneficial to patient care. Furthermore, 71 out of 89 respondents (80%) also agreed that using SDs at work assisted them in solving their personal issues.

### Opinion and Attitudes Toward Using Smart Devices and the Need for a Code of Conduct

A total of 40 out of 91 respondents (44%; as shown in Table 7) indicated that patients feel *positive* when they see the ED providers using their SDs during their stay in the ED for *clinical purposes*, whereas 20 out of 91 (22%) responded that the patients feel *negative* and 31 out of the 91 (34%) answered *neutral*. Furthermore, 67 out of 91 respondents (74%) responded that the patients feel *negative* when they see the ED providers using their SD during their stay in the ED for *nonclinical purposes*.

Furthermore, 52 out of 91 respondents (57%) felt *neutral* toward seeing fellow colleagues using their SD during their ED shift for nonclinical purposes, whereas 34 of them (37%) felt *negative* and only 5 respondents (6%) felt *positive*.

The opinion was split with regard to medical administrators establishing a code of conduct for the use of SDs to minimize unnecessary distraction during ED shifts, with 51 out of 91 respondents (56%) disagreeing and 40 of them (44%) being in agreement (Table 8).

### Smart Device Use Addiction Score

A total of 36 out of 90 respondents (40%) were found to be significantly addicted to their SDs (Table 9), with 62 out of 91 (68%) reaching for their SDs first thing in the morning and 32 out of 90 respondents (36%) feeling the need to cut down on the use of their SDs. Interestingly, 73 out of 91 respondents (80%) did not feel guilty about their overuse of the SDs at work nor felt annoyed when people criticized their use of the SDs (Table 10).

**Table 7:** Perceptions toward smart device use in the emergency department.

Perceptions toward smart device (SD) use in the emergency department (ED)		n (%)
**How do you think patients feel when they see the ED providers using their SD during their stay in the ED for clinical purposes? (N=91)**	
	Positive	40 (44)
	Negative	20 (22)
	Neutral	31 (34)
**How do you feel about fellow colleagues using their SD during their ED shift for nonclinical purposes? (N=91)**	
	Positive	5 (6)
	Negative	34 (37)
	Neutral	52 (57)
**How do you think patients feel when they see the ED providers using their SD during their stay in the ED for nonclinical work–related purposes (check/send personal emails to friends or family)? (N=91)**	
	Positive	2 (2)
	Negative	67 (74)
	Neutral	22 (24)

**Table 8:** Opinion and attitudes toward using smart devices in the emergency department and the need for a code of conduct.

The need for a code of conduct		n (%)
**Do you think that medical administrators should establish a code of conduct for the use of smart devices to minimize unnecessary distraction during emergency department shifts? (N=91)**	
	Yes	40 (44)
	No	51 (56)

**Table 9:** Smart device use addiction score.

Smart device use addiction score—the modified *Cut Down, Annoyed, Guilty and Eye Opener alcohol use disorders screening test* CAGE tool (N=90)		n (%)
**Respondents are** **clinically addicted to using** **smart devices**	
	Yes	36 (40)
	No	54 (60)

**Table 10:** Smart device use addiction score details.

Smart device use addiction score details—the modified *Cut Down, Annoyed, Guilty and Eye Opener alcohol use disorders screening test* CAGE tool		n (%)
**Do you reach for your smart device first thing in the morning?**	
	Yes	62 (68)
	No	29 (32)
**Have you ever felt you needed to cut down on use of your smart device?**	
	Yes	32 (36)
	No	58 (64)
**Have people annoyed you by criticizing your use of your smart device?**	
	Yes	18 (20)
	No	73 (80)
**Have you felt guilty about your overuse of your smart device at work?**	
	Yes	18 (20)
	No	73 (80)

### Predictors of Making an Error

The descriptive analysis of the health care providers making an error as a result of being distracted by their SDs was carried out (Multimedia Appendix 1).

Consistently, the acknowledgment of making an error was higher among the practitioners with more than 10 years of experience (3/9, 33%) compared with 1 out of 9 (11%) with 6 to 10 years of experience. The difference between the 2 groups was statistically significant (*P*=.049).

Interestingly, all the 15 respondents (100%) who acknowledged making an error used their SDs for personal matters during their ED shift (*P*=.04). In addition, out of those who acknowledged making an error, 3 out of 15 (20%) did acknowledge missing information when using their SDs during their ED shift (*P*=.03).

Furthermore, 10 out of 14 (71%) respondents who acknowledged making an error supported the need to implement a code of conduct to regulate the use of SDs in the ED (*P*=.03).

The research team developed a stepwise multivariate logistic regression model (model fitness=0.91) to help define the predictors of making an error because of using SDs in the ED (Table 11). The model shows that the respondents above 40 years of age were 5 times more likely to acknowledge making an error because of using SDs in the ED (*P*=.04), and those who missed information while using their SDs were 11 times more likely to commit an error (*P*=.02).

**Table 11:** Stepwise multivariate logistic regression of predictors of making error. Variables included in the stepwise regression model were with the following covariates: age (reference: <30 years); gender (reference: female); job (reference: medical student); missed information of using my smart device; used for personal purposes during your shift in the emergency department; addiction score.

Making error^a^ (reference: no)	Statistical values
	Odds ratio (95% CI)	*P* value
Age (≥40 years)	5.03 (1.09-23.15)	.04
Missed information of using my smart device	10.84 (1.49-78.91)	.02

## Discussion

### Principal Findings

This study revealed that the majority of the health care providers who responded to the survey did use an SD during their work shift and that a sizable proportion of respondents reported one of their colleagues or themselves getting distracted because of using SDs at the ED. Using a regression model, the analysis done by the research team shows that being above 40 years of age (*P*=.04) and missing information because of using SD (*P*=.02) are major predictors of committing an error during the work at the ED.

Findings of this study concur with those of other studies that revealed that SDs were being abundantly used in health care settings [19] and that the health care providers were aware of the medical applications [5]. In addition, the surveyed providers did hold positive views on the use of SDs with the majority stating that SDs enabled better team coordination, cohesion, and patient care. This study established that all health care providers used SDs during work shifts despite working in one of the busiest ED settings in the country. Such uses are not restricted to clinical applications that potentially support patient care, but also extend to personal uses in care settings. The propensity and the positive views on the use of SDs in patient care settings suggest that this is not a passing trend but rather one that is integrated into the health care practice environments nowadays. Health care practitioners are very much accustomed to using their SDs and hold positive views of such use.

However, the positive attitude expressed by the ED care providers is counterbalanced by some disconcerting findings. Although our results are based on self-reported distraction, the findings are in line with studies that have shown that the use of cell phones during functions that require cognitive focus increases reaction time, lowers performance, and reduces situational awareness through *inattentional blindness* to surrounding activities [20]. This distraction potential coupled with the growing body of literature on the addictive potential of SDs with reported addiction rates ranging between 5% to 65% are concerning within a health care context. In fact, 55% of the respondents in our study reported one of their ED colleagues making an error or a near miss related to SD usage. However, respondents fell short in acknowledging making an error or a near miss themselves (15/93, 16%). Such acknowledgment of medical errors and near misses is likely to be an underestimate of the actual rate. This is because it is difficult for health care providers to acknowledge their own medical errors owing to concerns about professional discipline and job security [21]. The propensity of errors and near misses caused by SD distraction is potentially detrimental to patient care and calls for immediate and affirmative action by the ED managers and stakeholders. The study makes it evident that using SDs is negatively affecting patient care outcomes irrespective of other reported positive effects of use.

### The Generation Gap

The findings of this study reveal that SD users tend to be younger and that they are more likely to accept the use of SDs in health care settings. Literature reports a generational gap with the use of SDs and technologies [22]. In our study, this gap has impacted the providers’ perceptions of SD use in the ED setting and their reporting of distractions as a result of using it, as well as their acknowledgment of error.

The results of the regression have clearly showed that ED providers aged 40 years or above are 5 times more likely to acknowledge making an error owing to the use of SDs in the ED. This could either be attributed to the older providers feeling less friendly toward technology that enhances technology-induced errors, or that they are just more open and honest about themselves. This is in concordance with studies that established that older people are relatively less likely to accept SDs, which could be because of the age-related decline in cognitive abilities required for the proper use of SDs [23,24]. In addition to the age effect, older adults are less experienced with SDs than younger adults [25] and have concerns over their security and privacy, which may impact their use of SD and may restrict them from trying new functions, especially in the absence of onsite assistance [25,26].

This generation gap has also impacted the answers toward the need for a code of conduct to regulate the use of SDs in the ED. The providers who are older than 30 years supported a code of conduct compared with their younger counterparts. This difference in the way respondents viewed the regulation measures is significant (*P*=.03) and should be addressed by the policy makers while designing those policies and protocols. In the health care context, the different aspects of perceived usability and ease of use are highly relevant to performing the different health care tasks, and thus engaging the relatively older providers in designing and implementing SD use protocols in health care settings would probably contribute to a better acceptance and adoption of mobile health care technologies in the hospital setting [27].

### The Effect of Smart Device-Driven Interruptions on Near Misses and Medical Errors

This study revisits the impact of using SDs on the quality of the health care service in a critically busy environment. Consistent with the fact that the increasing popularity of SD use in clinical settings may lead to increased distractions [6], the majority of respondents in this study have reported themselves and others getting distracted as a result of using SDs at the ED. This is highly relevant in the ED setting which demands that health care providers stay consistently focused on their jobs. What compounds the challenge is that using SDs in the clinical settings is exclusive not only to clinical applications, but also for personal purposes which could distract providers from vital patient care operations [28].

The fact that providers who acknowledged missing information because of using their SDs were 11 times more likely to acknowledge an error is quite disconcerting and requires quick action. This directly links the SD distractions to patient safety and poses serious questions on the potential negative effects of using such devices in similar busy settings. The literature is rich in examples of mobile device distractions proving detrimental to patient health and service quality, including procedural failures and decrease in care quality because of distractions by SD in clinical settings [6]. This has led the Emergency Care Research Institute (ECRI) to mention *caregiver distractions from SD and other mobile devices* as one of the top 10 hazards for 2013 [29].

The effect of distractive use of SDs in the clinical settings could be overcome by considering the human and technical factors which could be potentially compromised. In addition to the required security and disinfection measures, certain regulation procedures can be adopted to minimize the negative effects of using SDs in the clinical settings and promote the culture of using such devices for clinical purposes in a systematic way that could positively contribute to the provision and delivery of the health care service.

### Regulating the Use of Smart Devices in the Health Care Settings

The use of SDs in health care settings is likely to grow in the future and thus necessitates proper regulating protocols [30]. Although SDs hold a lot of potential benefits to health care, it is critical to consider the potential harm to the process quality and thus there is need to regulate the use of SD in health care settings [30]. With around 68% of the respondents reaching out to their SDs first thing in the morning, the results of this study confirm such an addictive behavior among the health care providers.

Regulating the use of SDs in emergency and other health care settings is not an easy task. Completely banning the use of SDs in health care institution is a difficult and highly unpopular option. In addition, it is important to recognize that imposing rules on using SDs for nonwork–related purpose would not be easy if the privately owned SDs are allowed within the clinical setting [5].

The analysis conducted by Gill and colleagues presents an interesting framework to regulate SD use in health care settings. The proposed framework aims to minimize distractions that are because of using SDs in the health care setting, keeping the patient health outcomes at the core. In addition to other measures, the framework allocates time to train the providers on security concerns and expectations, establishes clear security measures over the institutional network, and regulates the use of nonwork–related applications [6].

One of the interesting suggestions would be to set the basis for such a regulation culture during the medical education journey. Previous research suggests that hospitals should set an agenda to raise awareness on the proper use of SDs and promote professional use [31].

Regulating the use of SDs in health care is not an easy task, and hospitals should not expect it to see positive outcomes on the short run. This process is challenging as it aims at addressing addiction to SDs. This calls for a concerted multistakeholder effort involving medical organizations, health care providers, educators, and industry as well to improve the integration of SDs in the daily medical practice in an efficient, reliable, and safe way [32].

### Limitations

The study has a number of shortcomings that are worth mentioning. First, despite the research team assuring respondents of the confidentiality of their responses, there remains a risk of a social desirability bias with health care providers potentially modifying their responses to reflect favorable SD use. Second, the cross-sectional nature of the study could only establish significant associations with causality requiring other types of study designs (eg, longitudinal). Third, the study questionnaire, which has been reviewed by an expert panel and pilot tested, did not undergo validation in terms of comparison with the gold standard assessment tool. Finally, the relatively low sample size may not have provided the required power to reveal significant differences. It is thus recommended that the study is replicated at a larger scale to unearth significant associations between variables.

### Conclusions and Future Implications

With the wide proliferation and use of SDs in health care settings, it is imperative to ensure the safety and wellbeing of patients, particularly in high intensity, high volume department such as the ED. This study sheds light on the critical effects of using SDs in EDs, including the predictors and causes of making an error and its potential consequences on the care process from the perspective of the health care providers. Irrespective of the positive role the SDs play in the health care process, the negative effects of their use still mandate proper regulation. This is an ethical mandate taking into consideration the important consequences such use may have on care processes and outcomes.

## Appendix

Multimedia Appendix 1 Descriptive analysis of the factors associated with making an error and/or acknowledging it.

## Abbreviations

CAGE: Cut Down, Annoyed, Guilty and Eye Opener alcohol use disorders screening test
ED: emergency department
IRB: Institutional Review Board
SD: smart device
