# Supplementary material for: The Use of Smart Devices by Care Providers in Emergency Departments: Cross-Sectional Survey Design
Source: JMIR Mhealth Uhealth. 2019 May 5;7(6):e13614. doi: 10.2196/13614 (PMC6592497; doi:10.2196/13614)
Supplement: Multimedia Appendix 1 [file mhealth_v7i6e13614_app1.pdf]

APPENDIX A

Table 7: Descriptive analysis of the factors associated with making an error and/or acknowledging it

Variables	Values	I have Made an Error as a resulting of using my smart device at the ED		P value (Significant values are marked with an asterisk)
		No N=78	Yes N=15	
**Gender**	Female	39 (51.3%)	5 (38.5%)	.39
	Male	37 (48.7%)	8 (61.5%)	
**Age**	<30	52 (66.7%)	7 (46.7%)	.04*
	30-39	19 (24.4%)	3 (20.0%)	
	≥40	7 (9.0%)	5 (33.3%)	
**Job**	Medical student	17 (21.8%)	5 (33.3%)	.17
	Nurse	29 (37.2%)	2 (13.3%)	
	Resident	19 (24.4%)	3 (20.0%)	
	Attending physician	13 (16.7%)	5 (33.3%)	
**Experience in years**	<5 years	14 (50.0%)	0 (0.0%)	.049*
	6 to 10 years	8 (28.6%)	1 (25.0%)	
	>10 years	6 (21.4%)	3 (75.0%)	
**Do you use a smart tablet**	Yes	25 (32.1%)	3 (20.0%)	.54
**Do you use a smart watch**	Yes	3 (3.8%)	0 (0.0%)	1.000
**Do you bring Smart device to ED**	Yes	78 (100.0%)	15 (100.0%)	NA
**Do you use any medical related applications on your smart device**	Yes	67 (85.9%)	13 (86.7%)	1.000
**Do you use Medication formulary/drug reference applications**	Yes	66 (85.7%)	12 (80.0%)	.69
**Do you use Clinical score systems/medical Calculator applications**	Yes	52 (62.5%)	10 (66.7%)	1.000
**Do you use Disease diagnosis/management applications**	Yes	53 (69.7%)	9 (60.0%)	.55

**Do you use Procedure documentation applications**	Yes	16 (24.6%)	4 (26.7%)	1.000
**Do you use Web access of applications**	Yes	66 (85.7%)	11 (73.3%)	.26
**Do you use Email access applications**	Yes	68 (90.7%)	14 (93.3%)	1.000
**Do you use Calendar applications**	Yes	58 (76.3%)	11 (73.3%)	.75
**Do you use Social applications**	Yes	49 (63.6%)	11 (78.6%)	.37
**Do you use Messaging applications**	Yes	66 (85.7%)	14 (93.3%)	.68
**Do you use your smart device for Phone calls**	Yes	57 (74.0%)	12 (80.0%)	.75
**Do you use your smart device for Clinical care related use in ED**	Yes	71 (91.0%)	15 (100.0%)	.59
**Do you use your smart device for the personal use during your shift in the ED**	Yes	59 (76.6%)	15 (100.0%)	.04*
**I have observed a trainee use their smart device during their ED shift**	Yes	75 (96.2%)	15 (100.0%)	1.000
**I have observed a nurse use their smart device during their ED shift**	Yes	69 (88.5%)	13 (86.7%)	1.000
**I have observed an attending physician use their smart device during their ED shift**	Yes	76 (97.4%)	14 (93.3%)	.41
**I have observed trainees use their smart devices during ED shifts for Clinical care related use**	Yes	68 (93.2%)	13 (92.9%)	1.000
**I have observed trainees use their smart devices during ED shifts for personal use**	Yes	65 (87.8%)	14 (100.0%)	.34
**I have observed attending use their smart devices during ED shifts for Clinical care related use**	Yes	63 (86.3%)	14 (93.3%)	.68

**I have observed attending use their smart devices during ED shifts for personal use**	Yes	66 (90.4%)	15 (100.0%)	.6
**I have observed nurses use their smart devices during ED shifts for Clinical care related use**	Yes	50 (67.6%)	6 (42.9%)	.12
**I have observed nurses use their smart devices during ED shifts for personal use**	Yes	63 (84.0%)	13 (86.7%)	1.000
**I have missed information as a result of using my smart device**	Yes	2 (2.6%)	3 (20.0%)	.03*
**Using the smart device has positively impacted my performance at work**	Yes	30 (41.7%)	3 (20.0%)	.12
**I have witnessed a trainee getting distracted as a result of using the smart device**	Yes	45 (58.4%)	13 (92.9%)	.01*
**I have witnessed a nurse getting distracted as a result of using the smart device**	Yes	40 (53.3%)	10 (66.7%)	.34
**I have witnessed an attending getting distracted as a result of using the smart device**	Yes	32 (43.2%)	11 (73.3%)	.03*
**Have any of your ED co-workers made an error as a result of using the smart device**	Yes	36 (46.2%)	15 (100.0%)	<0.001*
**Using smart devices allowed better coordination among the healthcare providers**	Yes	57 (78.1%)	13 (100.0%)	.12
**Using smart devices improved ED cohesion teamwork**	Yes	41 (58.6%)	5 (35.7%)	.12
**Using smart devices improved patient safety**	Yes	43 (63.2%)	4 (28.6%)	.02*

**Using smart devices is beneficial to patient care**	Yes	59 (78.7%)	11 (78.6%)	1.000
**Using smart devices assisted in solving my personal issues**	Yes	62 (83.8%)	9 (64.3%)	.13
**Using smart devices reduces my stress**	Yes	56 (77.8%)	8 (57.1%)	.18
**Do you support the need for a code of Conduct**	Yes	30 (39.5%)	10 (71.4%)	.03*
**My feeling about colleagues when use devices for non-clinical purposes**	Negative<br>Neutral<br>Positive	26 (34.2%)<br>46 (60.5%)<br>4 (5.3%)	8 (57.1%)<br>6 (42.9%)<br>0 (0.0%)	0.3
**Patient feelings feel when see the providers using smart devices for non-clinical purposes**	Negative<br>Neutral<br>Positive	54 (71.1%)<br>22 (28.9%)<br>0 (0.0%)	13 (92.9%)<br>0 (0.0%)<br>1 (7.1%)	.01*
**Patient feelings when see the providers using smart devices for clinical purposes**	Negative<br>Neutral<br>Positive	17 (22.4%)<br>27 (35.5%)<br>32 (42.1%)	3 (21.4%)<br>4 (28.6%)<br>7 (50.0%)	.93
**Using smart devices (the adapted CAGE tool)**				
**First thing in the morning**	Yes	49 (64.5%)	12 (85.7%)	.21
**Felt to cut down**	Yes	23 (30.7%)	8 (57.1%)	.07
**People annoyed criticizing**	Yes	13 (17.1%)	4 (28.6%)	.46
**Felt guilty**	Yes	12 (15.8%)	5 (35.7%)	.13
0 of the above<br>At least one of the above<br>At least two of the above<br>At least 3 of the above<br>All of the above	Yes<br>Yes<br>Yes<br>Yes<br>Yes	18 (23.7%)<br>30 (39.5%)<br>18 (23.7%)<br>9 (11.8%)<br>1 (1.3%)	0 (0.0%)<br>6 (42.9%)<br>3 (21.4%)<br>3 (21.4%)<br>2 (14.3%)	.03*
Insignificant addiction score (average <2)<br>Significant addiction score (average ≥2)	Yes<br>Yes	48 (63.2%)<br>28 (36.8%)	6 (42.9%)<br>8 (57.1%)	.15
